# Examining Ferromagnetic Materials Subjected to a Static Stress Load Using the Magnetic Method

**DOI:** 10.3390/ma14133455

**Published:** 2021-06-22

**Authors:** Tomasz Chady, Ryszard Łukaszuk

**Affiliations:** 1 Faculty of Electrical Engineering, West Pomeranian University of Technology in Szczecin, Sikorski St. 37, 70-313 Szczecin, Poland; 2 Independent Researcher, Sikorski St. 37, 70-313 Szczecin, Poland; tramwajarz.szczecinski@gmail.com

**Keywords:** nondestructive evaluation, magnetic methods of testing, NDT

## Abstract

This paper discusses the experimental examination of anisotropic steel-made samples subjected to a static stress load. A nondestructive testing (NDT) measurement system with a transducer, which enables observation of local hysteresis loops and detection of samples’ inhomogeneity, is proposed. Local hysteresis loops are measured on two perpendicular axes, including one parallel to the rolling direction of the samples. The results confirm that the selected features of the local hysteresis loops provide important information about the conditions of ferromagnetic materials. Furthermore, it is shown that the selected parameters of the statistical analysis of the achieved measurements are beneficial for evaluating stress and fatigue changes induced in the material.

## 1. Introduction

Steel is susceptible to the harmful effects of certain external environmental factors. For this reason, it is necessary to subject steel products to examination at the stage of both production and operation. If the internal and external structure of the object must remain intact, nondestructive testing (NDT) is performed. Detection of small inhomogeneities in the material allows us to observe degradation at an early stage, reducing the possibility of a catastrophic failure and alternative repair costs. The good electrical conductivity and high permeability of steel create possibilities to detect discontinuities in its structure using electromagnetic methods of NDT. The electromagnetic methods have high sensitivity, so apart from detecting a defect, it is also possible to pinpoint its location and assess its dimensions. In the case of steel-made sheets rolled in the direction opposite to the grain orientation, magnetic anisotropy is a particular obstacle during an examination. In addition, anisotropy can also be induced by stress. This results in the need for the inspection to be carried out in two orthogonal directions of the material.

Ferromagnetic materials can be tested using several electromagnetic methods of NDT:

▪ the magnetic flux leakage method is based on observation of the magnetic flux distribution over the material surface [1]. The primary magnetic field source causes a magnetic flux in the material. A barrier to the secondary flux is any inhomogeneity in the material structure that has a significant reluctance value [2]. The flux leakage method allows us to assess the tested object’s surface and subsurface inhomogeneity [3]. The main advantages are high sensitivity, easiness of signal acquisition, and the possibility of automation [2,4]. However, this method also has some disadvantages, including sensitivity to material impurities and the need to magnetize the object [5];
▪ the magnetic particle inspection method allows for the detection of both surface and subsurface heterogeneities [6]. First, the sample is exposed to an external magnetic field, whereby magnetic powder particles can be placed on the outer surface of the sample in two ways: during the magnetization or after switching the magnetic field source off. The magnetic flux dispersing on the inhomogeneities appears on the material’s surface and changes the distribution of the particles [7]. The resulting image contains foci of particles that indicate the material heterogeneities [8]. Instead of magnetic powder, a suspension liquid can also be used to enhance the inspection sensitivity. Nowadays, apart from traditional indicators such as magnetic powders and suspension liquids, GMR or Hall sensors are also applicable to the inspection. The magnetic particle method is a quick, inexpensive, and relatively uncomplicated inspection method that gives immediate indications of surface and near-surface defects.
▪ the eddy current testing method is based on observing the flow path of the induced currents in the examined material [9]. The excitation magnetic field source induces eddy currents in the material. Disturbances in eddy current flow caused by inhomogeneities become apparent in the resultant field [10]. The advantages of testing with eddy currents are the high efficiency of detecting even the most minor defects, no need for direct access, and the penetration of many layers of material [11,12]. The main disadvantage of this method is that it only detects defects located not too deep under the surface due to the skin effect, which is especially strong in the case of ferromagnetic materials [13];
▪ the Barkhausen noise method relies on observing the magnetization process, which causes the dipoles to rotate [14,15,16]. If the material contains inhomogeneities, the process of domain wall shifting will be disrupted. As a result, sudden magnetization changes induce voltage pulses, which become apparent and can be observed as Barkhausen noise [17,18]. The Barkhausen noise testing method may be beneficial in some cases because of its low cost, high reliability, and simplicity [19]. The method is especially useful for stress monitoring. However, it has also some drawbacks, such as limited sensitivity resulting from thermal effects [20];
▪ the 3MA is an approach that combines features of four NDT methods: the Barkhausen noise, eddy current, incremental permeability, and harmonic analysis of magnetic field strength methods. Using several methods simultaneously reduces the likelihood of inconclusive inspection results [21];
▪ the magnetic memory method is based on the measurement of the residual magnetization, which appears in the material under the influence of a stress load or external geomagnetic fields [22]. The residual magnetization is recorded using sensors and then analyzed to assess defects [23]. The advantages of this method are the possibility to detect failures at an early stage, the lack of a need to provide an external magnetic field, and its simplicity [24]. A significant drawback is that it generally can be used only as an auxiliary method because of its low accuracy [24];
▪ the hysteresis loop observation method is another method aimed at localizing stress and heterogeneities. The microstructure of the ferromagnetic material strongly affects the hysteresis loop shape [25]. If the tested object is subjected to stress, the coercivity field and remanence induction values change due to the displacement of the dipoles separated by Bloch walls [26,27].

This work presents an examination of steel-made samples subjected to a static tensile stress load. The raw data acquired during the observation of the local hysteresis loops in two perpendicular directions were normalized, visualized, and analyzed using an approach based on statistics.

## 2. Materials and Methods

The subject of the examination consisted of specimens made of SS400 (JIS3101 standard) steel with an applied tensile stress. SS400 is one of the most commonly used hot-rolled general structural carbon and low-alloy steels designated for such structures as bridges, ships, and rolling stocks.

The mechanical properties of SS400 are as follows [28]: yield strength, 350 MPa; elastic modulus, 209 GPa; Poisson’s ratio, 0.29; and chemical composition (wt), C—0.148%, Mn—0.458%, Si—0.213%, S—0.018%, P—0.012%, and Fe—bal.

**Figure 1:**
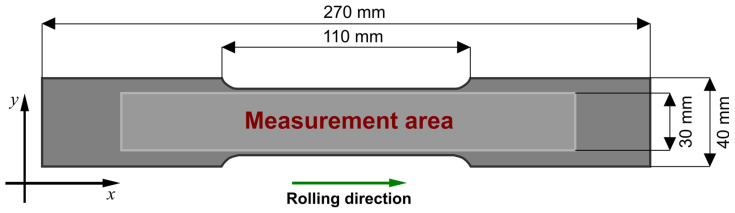
Shape and dimensions of the samples used in the experiments.

The shape and dimensions of the SS400 specimens used in the experiments are shown in Figure 1. They are different from the standard tensile test specimens because a sufficient area for measurements has to be provided for the relatively large transducer. The samples were manufactured using a water jet cutting machine to avoid sharp edges, eliminate the heat-affected zone, and minimize introduced stress.

Before the experiment, each specimen (except the reference one, named S00) was subjected to a static tensile stress load at ambient temperature. The applied stress in the longitudinal direction (*x*-axis) was coincident with the rolling direction of the specimen.

**Figure 2:**
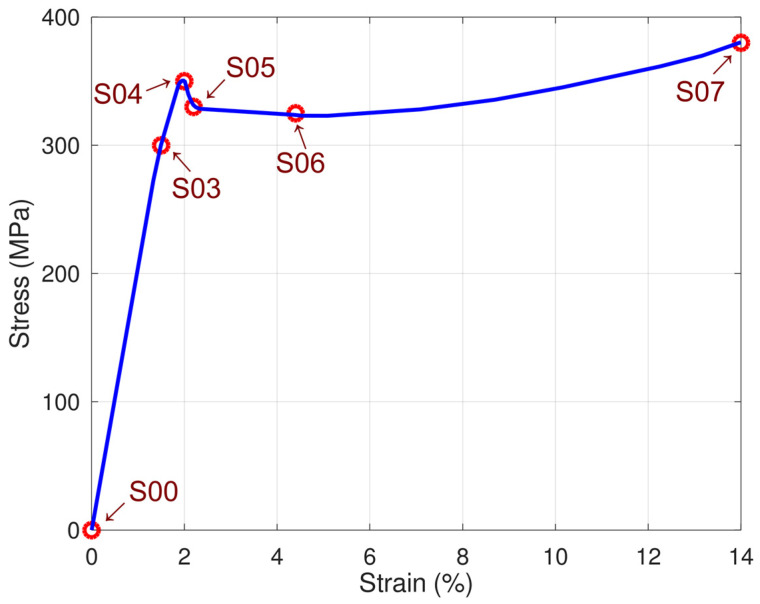
The stress–strain curve for the examined SS400 specimens.

Before the inspection, five samples (named S03, S04, S05, S06, and S07) were prepared and loaded using a standard Instron system. For each sample, the tensile stress test was stopped for different strain values (Figure 2). The residual strain ε was between 0.7% and 14%, while the maximum tensile stress was 389 MPa. Samples S04 and S05 were stress-loaded, respectively, up to the yield point (strain ε = 2%) and over it (strain ε = 2.4%). For illustrative purposes, all samples are marked on a single stress–strain curve, which is shown in Figure 2.

Protection of the top specimen’s surface from mechanical damage and the transducer from rupture of the measurement windings was provided by a polytetrafluoroethylene (PTFE) tape with a thickness of 0.2 mm. The samples were placed below the transducer one by one, attached to the scanning system’s head, and the transducer was aligned with the *x*-axis direction (Figure 1). During the measurements, the scanning system’s head was moved to the upper left corner of the area to be examined. The transducer moved along the *x*-axis at a 200 mm distance and stopped for 400 ms every 0.5 mm to read and record signal values. The procedure was repeated by analogy 25 times for other transducer positions (every 1 mm) varying along the *y*-axis. In the subsequent part of the inspection, the transducer was aligned with the *y*-axis direction (Figure 1) by rotating it 90 degrees clockwise. The measurement procedure with the rotated transducer was analogous to the measurements performed previously. The signal values were stored on a computer for further processing.

The main objective was to observe changes in the hysteresis loop caused by the stress applied to the samples before the inspection. The magnetization during the measurements was carried out twice on two perpendicular axes (one axis was parallel to the rolling direction).

**Figure 3:**
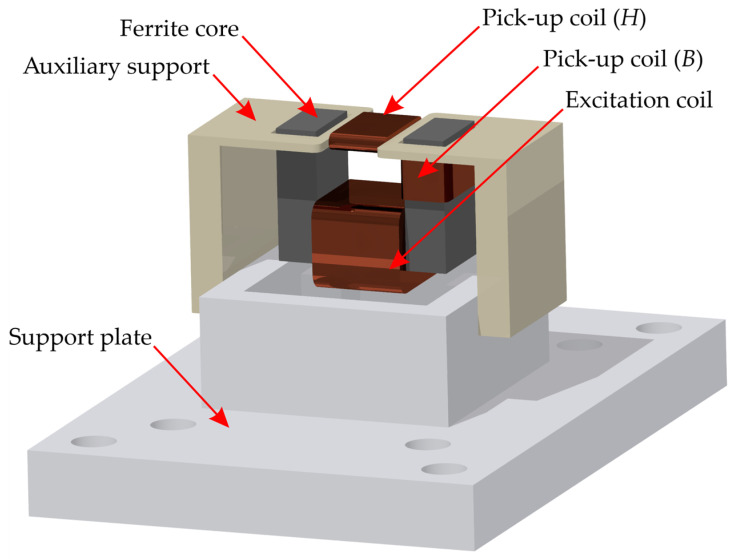
Transducer model.

**Figure 4:**
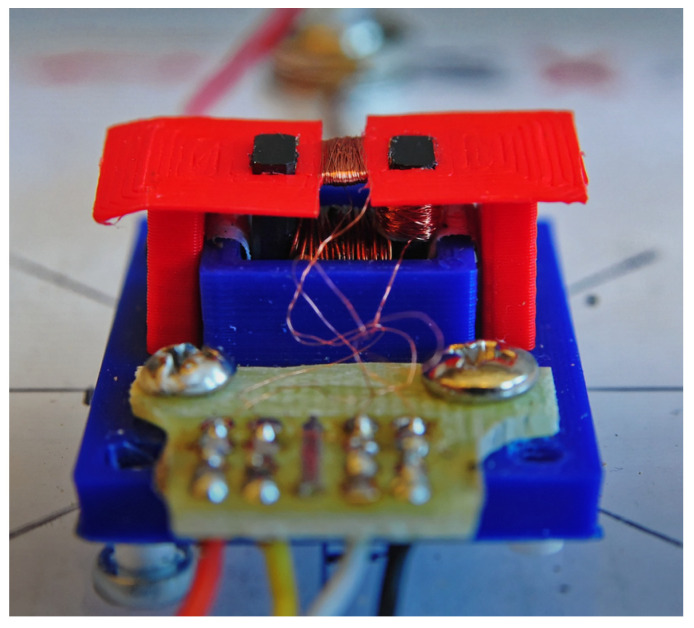
Photo of the transducer.

Examination of the samples was carried out using a transducer. The transducer consists of a support plate, an auxiliary support, and three coils. The stillage carries a u-shaped ferrite core with two pick-up coils, while the excitation coil is attached to the auxiliary support (Figure 3 and Figure 4). Selected transducer parameters are shown in Table 1.

**Figure 5:**
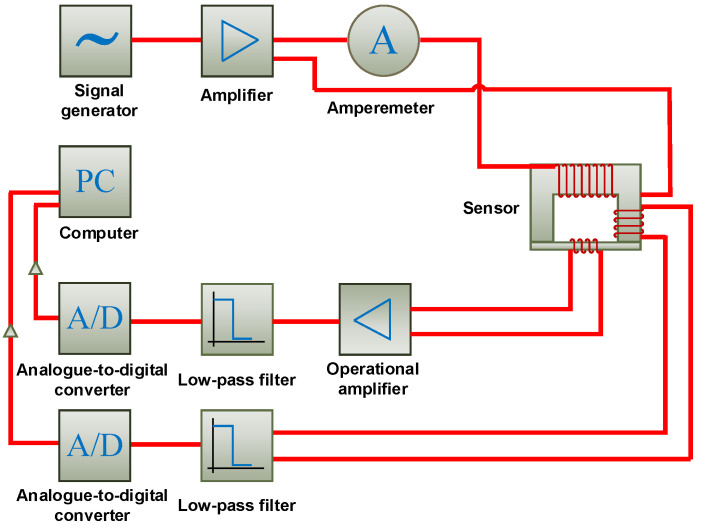
A block scheme of the measuring system.

The transducer works in the system shown in Figure 5. The system consists of a scanning device, a signal generator, A/D signal converters, amplifiers, the transducer, and a control computer. Parameters of the requested excitation signal (amplitude and frequency *f*_E_ = 4.4 kHz) are sent to the signal generator, which provides a relevant voltage signal to the power amplifier. The excitation frequency was selected considering the influence of noise, the level of induced voltages in the pick-up coils, and the results of preliminary experiments carried out with the SS400 sample. Subsequently, the boosted signal supplies an excitaon coil of the transducer. This coil induces a primary magnetic field, which flows through the transducer’s ferromagnetic core and two pick-up coils (*B* and *H*) and penetrates the tested material. Then, the voltage signal from a field-sensing pick-up coil (*H*) reaches the instrumentation amplifier and, after being modified by a second-order Butterworth low-pass filter with a cutoff frequency of 50 kHz, enters an analog-to-digital converter. A signal from a flux-sensing pick-up coil (*B*) is passed directly to the second-order Butterworth low-pass filter with a cutoff frequency of 50 kHz and digitalized. Eventually, both voltage signal values are saved in the computer’s memory for further analysis. Detailed information about the measurement system is provided in Table 1.

The raw data collected from the two-dimensional scanning of the samples were processed using dedicated software. First, the data were normalized and corrected by removing distortions. Subsequently, the values of the hysteresis loop parameters were calculated for both axes, and graphs were plotted showing the relative change in a given parameter as a function of the coordinates of the point from the measurement area (Figure 1).

The following calculations were done to assess the condition of the examined samples. First, every single magnetic loop parameter in two perpendicular magnetization directions (1) was calculated.

(1) $$\Delta k_{Amax}\left(x,y\right)=k_{Amax}\left(x,y\right)-k_{Amax}\left(x_{0},y_{0}\right),$$

where: k—the chosen magnetic loop parameter, A—the selected magnetization direction, x,y—the measurement area point’s coordinates, $k_{Amax}\left(x,y\right)$—the maximum values of the chosen magnetic loop parameter, and $k_{Amax}\left(x_{0},y_{0}\right)$—a mean value calculated from measurements achieved for points located within a distance of 2 mm from a starting point having the coordinates $\left(x_{0},y_{0}\right)$. The starting point was located in the broader part of the sample, where the stress level was significantly lower. A similar point of construction will have to be selected as the reference point of the real tested structures.

Afterwards, normalized relative changes in the maximum (2) and minimum (3) magnetic induction, maximum (4) and minimum (5) magnetic field strength, as well as the area of the hysteresis loop (6) were calculated.

(2) $$K_{A1}\left(x,y\right)=\frac{\Delta B_{Amax}\left(x,y\right)}{\max\left(\left|\Delta B_{Amax}\left(x,y\right)\right|\right)}$$

(3) $$K_{A2}\left(x,y\right)=\frac{\Delta B_{Amin}\left(x,y\right)}{\max\left(\left|\Delta B_{Amin}\left(x,y\right)\right|\right)}$$

(4) $$K_{A3}\left(x,y\right)=\frac{\Delta H_{Amax}\left(x,y\right)}{\max\left(\left|\Delta H_{Amax}\left(x,y\right)\right|\right)}$$

(5) $$K_{A4}\left(x,y\right)=\frac{\Delta H_{Amin}\left(x,y\right)}{\max\left(\left|\Delta H_{Amin}\left(x,y\right)\right|\right)}$$

(6) $$K_{A5}\left(x,y\right)=\frac{\Delta P_{A}\left(x,y\right)}{\max\left(\left|{\Delta P}_{A}\left(x,y\right)\right|\right)}$$

where: A—the magnetization direction, x,y—the measurement area point’s coordinates, $\Delta B_{Amax}\left(x,y\right)$—the relative change in the maximum magnetic induction, $\Delta B_{Amin}\left(x,y\right)$—the relative change in the minimum magnetic induction, $\Delta H_{Amax}\left(x,y\right)$—the relative change in the maximum magnetic field strength, $\Delta H_{Amin}\left(x,y\right)$—the relative change in the minimum magnetic field strength, and $\Delta P_{A}\left(x,y\right)$—the relative change in the local hysteresis loop’s area.

The next step concerned performing statistical analysis to assess the conditions of the material (after the stress loading). Initially, frequency histograms were plotted for the hysteresis loop parameters measured for all samples. Then, the focus was on performing statistical analysis of the data.

## 3. Results and Discussion

The measurements were done according to the procedure described in Section 2. As a result of these measurements, a set of signals was obtained for each sample, necessary to plot the hysteresis loop measured for magnetization in the *x-* and *y*-axis directions. The signals were achieved for all positions (*x*_i_, *y*_i_) over which the transducer was moved. Due to the design of the transducer and the shape of the samples, in the case of magnetization in the *y*-axis, the shift range in the *y*-axis was slightly smaller than the magnetization in the *x*-axis direction.

**Figure 6:**
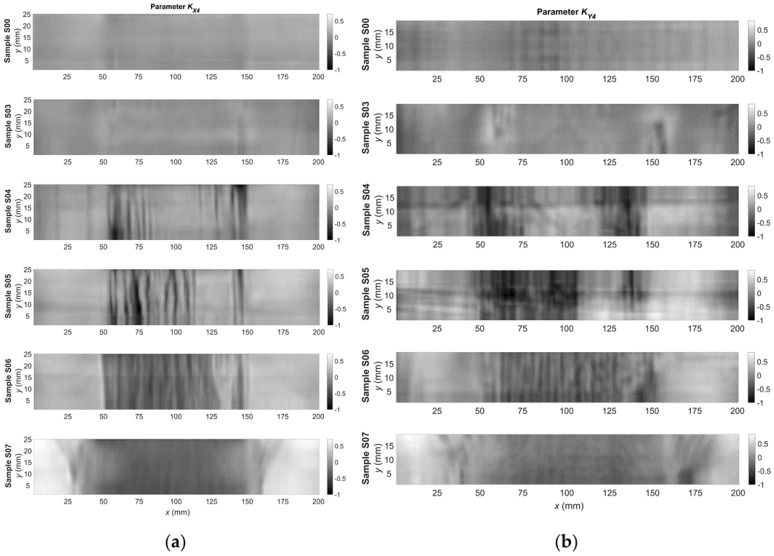
Relative change in the $K_{A4}$ parameter as a function of the coordinates of the point from the measurement area for the: (**a**) *x*-axis direction; and (**b**) *y*-axis direction.

**Figure 7:**
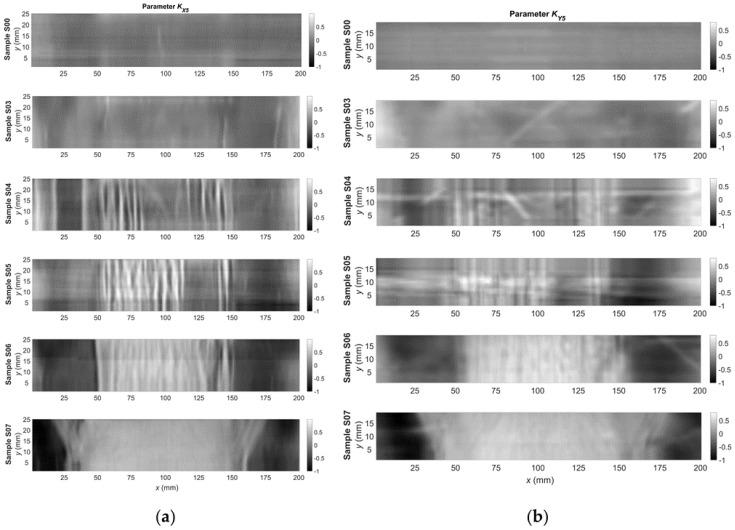
Relative change in the $K_{A5}$ parameter as a function of the coordinates of the point from the measurement area for the: (**a**) *x*-axis direction; and (**b**) *y*-axis direction.

Next, the parameters defined by Equations (2)–(6) were calculated using the signals acquired for each of the measuring points. Examples of two-dimensional plots of these parameters are presented in Figure 6 and Figure 7. The plots show changes in the parameter’s value over the surface sample. It is possible to generally observe a good correlation between the quantities measured on both orthogonal axes, but also significant differences are visible. It allows for a hypothesis that these signals are complementary to each other. By analyzing the signals for samples S00, S03, and S04, it can be observed that the signal value increases significantly and then decreases in the case of S06 and S07. Similar trends occur both for the parameters *K_A_*_4_ (Figure 6) and *K_A_*_5_ (Figure 7).

Despite the visible trends, assessing the load condition of the sample directly based on the measured signals is complex and may lead to ambiguity, for example, when using only the maximum value of the parameter. For this reason, attempts were made to statistically analyze the determined parameters to become independent from random changes in the signal value and take into account only the general trends.

**Figure 8:**
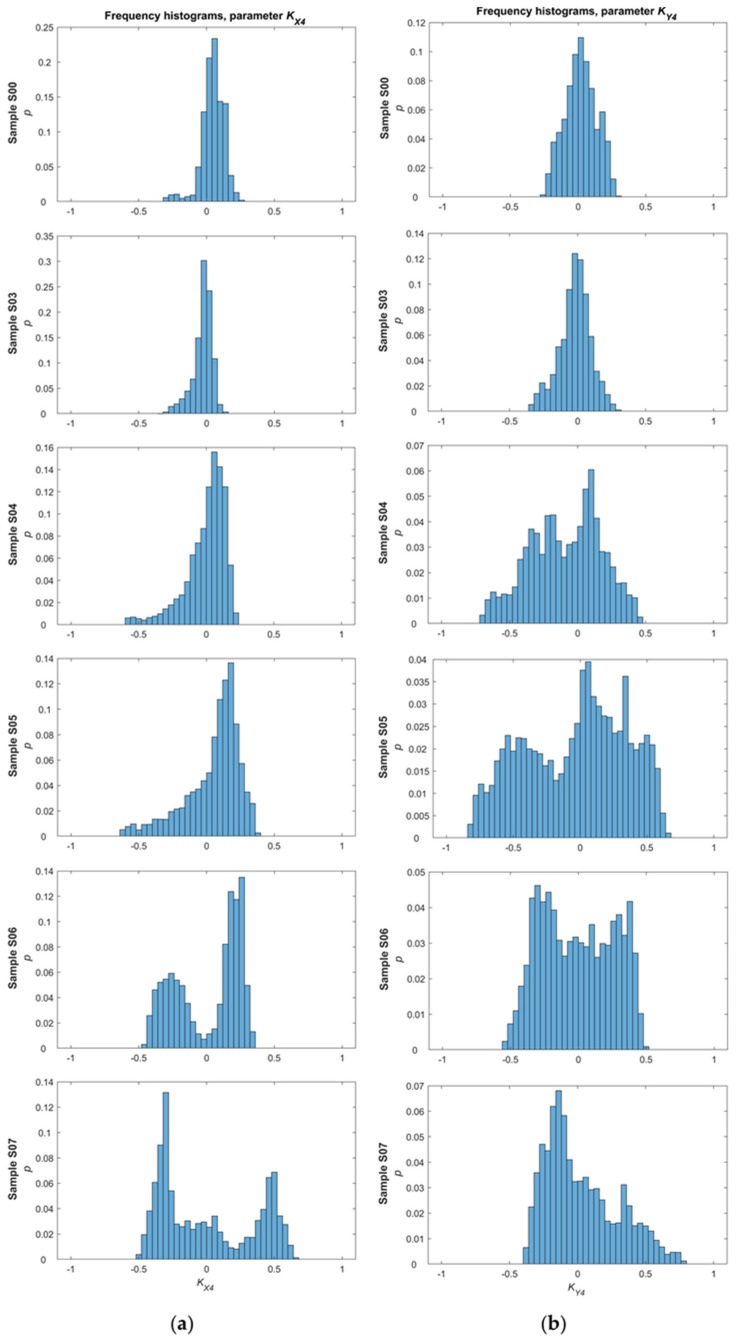
Frequency histograms of the $K_{A4}$ parameter for the: (**a**) *x*-axis direction; and (**b**) *y*-axis direction.

**Figure 9:**
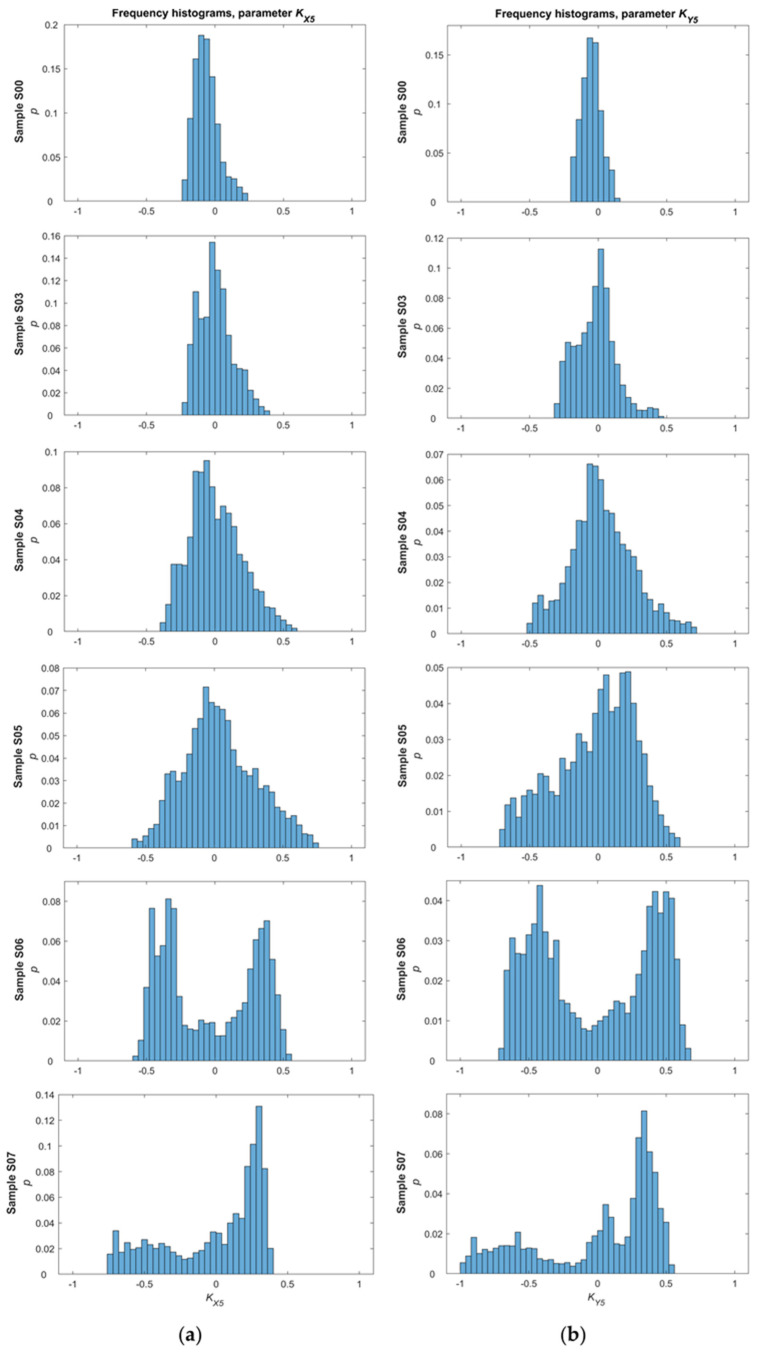
Frequency histograms of the $K_{A5}$ parameter for the: (**a**) *x*-axis direction; and (**b**) *y*-axis direction.

In the first phase of the analysis, a frequency histogram was prepared for each of the parameters determined from measurements carried out for a single sample and one direction of magnetization (Figure 8 and Figure 9). In the beginning, frequency histograms were subjected to normalization, relying on reducing the influence of interference signals. Then, the focus was on performing statistical analysis of the data by calculating and visualizing the following values: the maximum and minimum value, expected value, median, mode, variance, standard deviation, kurtosis, and histogram intervals. The frequency histograms achieved for samples with various levels of introduced stress show significant differences. For example, in the case of the *K_X_*_4_ parameter (Figure 8a), interesting changes in skewness and distribution modality can be observed. The distribution is almost symmetrical and unimodal for the undeformed sample, an evident skewness appears starting from sample S03, and from sample S06 the distribution becomes bimodal. The reason for the transition from a unimodal to a bimodal distribution is the increasing level of stress and dislocations in the internal structure of successive samples. In the second part of the analysis, statistical values for the *K_A_*_4_ and *K_A_*_5_ parameters on both axes were computed as already mentioned. However, some statistical values, such as variance, expected value, standard deviation, and kurtosis, could not eventually be taken into account due to non-monotonic changes and a problem with unequivocal identification of material conditions.

**Figure 10:**
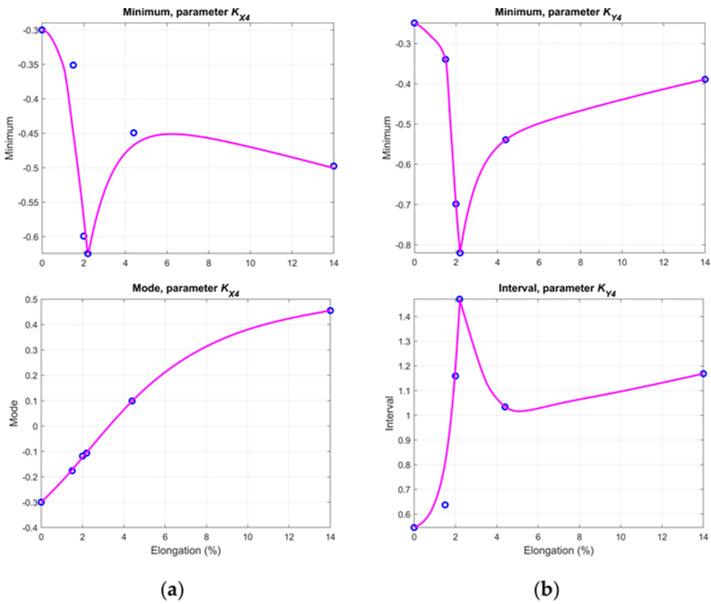
Chosen statistical values of the $K_{A4}$ parameter for the: (**a**) *x*-axis direction; and (**b**) *y*-axis direction.

**Figure 11:**
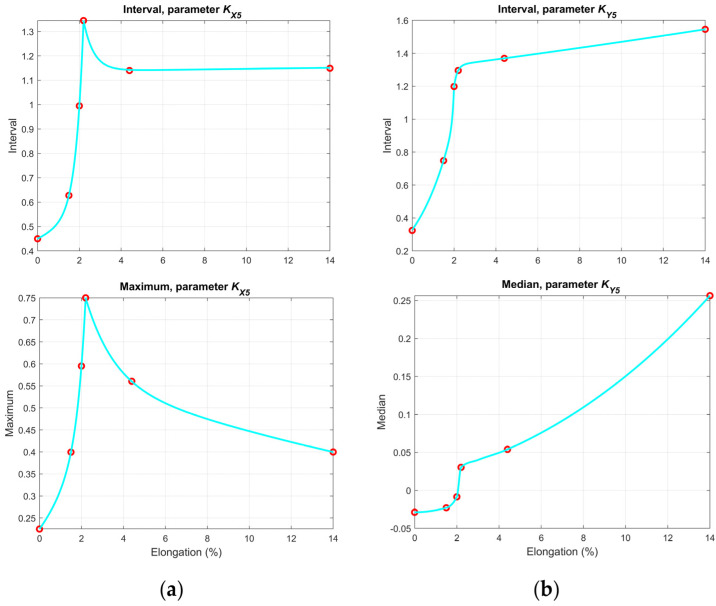
Chosen statistical values of the $K_{A5}$ parameter for the: (**a**) *x*-axis direction; and (**b**) *y*-axis direction.

Figure 10 and Figure 11 contain plots of selected statistical values, which create the best opportunity to evaluate the conditions of a given sample.

As shown in Figure 10, the minimum values of both the *K_X_*_4_ and *K_Y_*_4_ parameters decrease from the undamaged sample S00 to sample S05. Around specimens S05 and S06, the stress level caused the yield point to be exceeded, which can also be observed in the graphs as an inflection of the curve. After passing the yield point and increasing the stress level, the values decrease (in the case of the *K_X_*_4_ parameter) or increase (in the case of the *K_Y_*_4_ parameter). Mode and interval curves allow for the state of the samples to be determined straightforwardly as well. Graphs related to *K_X_*_5_ (Figure 11) indicate that the interval value increases up to the S05 sample and then the curves bend due to the growing number of inhomogeneities. Particularly beneficial are the interval and median values of the *K_Y_*_5_ parameter, enabling the identification of the sample’s condition unequivocally (both before and after yield).

The main goal of all the tests and analyses was to find the parameters of the measured signals that would allow for the assessment of the condition of the tested structure in terms of the stresses they were subjected to. An important guess was to choose such individual parameters or groups of parameters that guarantee assessment in both the elastic and plastic regions. The use of histograms and statistical features made the results independent of randomly changing signals caused by disturbances and other external factors, such as surface unevenness. Therefore, the parameters presented in Figure 10 and Figure 11 create a good chance that the assumed goals have been achieved. One of the important conclusions is the need to carry out measurements in at least two directions (parallel and perpendicular to the expected direction of stresses). A good example is the *K_A_*_5_ parameter measured in the *y*-axis direction (Figure 11b), which allows for an unambiguous identification of the state. Such parameters are crucial for building an automatic identification system in the future, which will use several of the presented parameters and the rough sets algorithm.

## 4. Conclusions

The achieved results allow us to conclude that the proposed approach based on nondestructive testing using observation of hysteresis loops and selected statistical analysis methods utilizing frequency histograms can be helpful to evaluate the condition of ferromagnetic materials subjected to a static stress load. Nevertheless, further research is needed to assess the usefulness of the statistical parameters and their applicability for testing other ferromagnetic materials (e.g., various construction steels). Based on the achieved results, it is recommended that not just one but several statistical parameters be used when assessing the state of a material under evaluation. In addition to the tests presented in this paper with the transducer aligned in the two perpendicular directions, additional measurements could also be performed for the transducer rotated at 45 degrees to the rolling axis. Such a methodology could provide additional information about the inhomogeneities in the material (e.g., higher sensitivity in the case of Lüders band detection). Therefore, we plan to develop an integrated transducer consisting of three directional sensors.

## Figures and Tables

**Table 1:** Transducer parameters.

Parameter Definition	Parameter	Value
Excitation current RMS value	$I_{e}$	400 mA
Frequency of the excitation	$f_{e}$	4.4 kHz
Low-pass filter cutoff frequency	$f_{l}$	50 kHz
Amplifier gain	*G*	30 dB
Ferrite core length	$l_{c1}$	10.5 mm
Ferrite core width	$l_{c2}$	5 mm
Ferrite core height	$l_{c3}$	8.2 mm
Pick-up coil (*H*) length	$l_{h1}$	3.2 mm
Pick-up coil (*H*) width	$l_{h2}$	2 mm
Pick-up coil (*H*) height	$l_{h3}$	4.75 mm
Pick-up coil (*H*) number of turns	$n_{H}$	90
Pick-up coil (*B*) length	$l_{b1}$	5 mm
Pick-up coil (*B*) width	$l_{b2}$	2.5 mm
Pick-up coil (*B*) height	$l_{b3}$	3 mm
Pick-up coil (*B*) number of turns	$n_{B}$.	100

## Data Availability

The data presented in this study are available on request from the corresponding author. The data are not publicly available due to a complicated structure that requires additional explanations.
